# Calycosin Suppresses Breast Cancer Cell Growth via ERβ-Dependent Regulation of IGF-1R, p38 MAPK and PI3K/Akt Pathways

**DOI:** 10.1371/journal.pone.0091245

**Published:** 2014-03-11

**Authors:** Jian Chen, Ruanling Hou, Xing Zhang, Yu Ye, Yong Wang, Jing Tian

**Affiliations:** 1 School of Basic Medical Sciences, Guilin Medical University, Guilin, China; 2 Department of Physiology and Neurobiology, Xinxiang Medical University, Xinxiang, China; 3 Department of Emergency, The First Affiliated Hospital of Guangxi Medical University, Nanning, China; 4 Department of Physiology, School of Basic Medical Sciences, Guilin Medical University, Guilin, China; Witten/Herdecke University, Germany

## Abstract

We previously reported that calycosin, a natural phytoestrogen structurally similar to estrogen, successfully triggered apoptosis of estrogen receptor (ER)-positive breast cancer cell line, MCF-7. To better understand the antitumor activities of calycosin against breast cancer, besides MCF-7 cells, another ER-positive cell line T-47D was analyzed here, with ER-negative cell lines (MDA-231, MDA-435) as control. Notably, calycosin led to inhibited cell proliferation and apoptosis only in ER-positive cells, particularly in MCF-7 cells, whereas no such effect was observed in ER-negative cells. Then we investigated whether regulation of ERβ, a subtype of ER, contributed to calycosin-induced apoptosis in breast cancer cells. The results showed that incubation of calycosin resulted in enhanced expression ERβ in MCF-7 and T-47D cells, rather than MDA-231 and MDA-435 cells. Moreover, with the upregulation of ERβ, successive changes in downstream signaling pathways were found, including inactivation of insulin-like growth factor 1 receptor (IGF-1R), then stimulation of p38 MAPK and suppression of the serine/threonine kinase (Akt), and finally poly(ADP-ribose) polymerase 1 (PARP-1) cleavage. However, the other two members of the mitogen-activated protein kinase (MAPK) family, extracellular signal-regulated kinase (ERK) 1/2 and c-Jun N-terminal kinase (JNK), were not consequently regulated by downregulated IGF-1R, indicating ERK 1/2 and JNK pathways were not necessary to allow proliferation inhibition by calycosin. Taken together, our results indicate that calycosin tends to inhibit growth and induce apoptosis in ER-positive breast cancer cells, which is mediated by ERβ-induced inhibition of IGF-1R, along with the selective regulation of MAPK and phosphatidylinositol 3-kinase (PI3K)/Akt pathways.

## Introduction

Epidemiological studies have shown that small increase in circulating estrogen may lead to breast cancer, which could be partially explained by estrogen-mediated tumor cell proliferation via binding to estrogen receptor (ER) [1], [2]. Accordingly, targeting the interaction between estrogen and ER-mediated signaling pathway is a promising therapeutic strategy in treating estrogen-dependent breast cancer. At present, plant-derived phytoestrogens are attracting attention for their structural and functional similarity with mammalian estrogen, by which phytoestrogens can elicit antiestrogenic or estrogen-like effects [3], [4]. Phytoestrogenic compounds are widespread in nature and subdivided into four main classes: isoflavones, stilbenes, coumestans and lignans [5]. Previously, we have demonstrated that calycosin, a main member of isoflavones, at relative high concentration induced apoptosis in human ER-positive breast cancer MCF-7 cells [6]. However, whether this anti-proliferation effect in breast cancer is ER-dependent remains unclear, not to mention the specific mechanism. Thus, in the present study, other than MCF-7 cells, another human ER-positive breast cancer cell line T-47D was also detected to provide more valuable information about calycosin-mediated regulation of ER signaling. In addition, ER-negative breast cancer cells MDA-231 and MDA-435 served as control to characterize the possible molecular mechanisms involved.

ER belongs to the steroid hormone receptor family and contains two subtypes, ER alpha (ERα) and ER beta (ERβ) [7]. It is found that the proportion of ERα-positive cells in estrogen-dependent breast cancers is higher than that of normal breast tissue, whereas the expression of ERβ is decreased, indicating an antagonistic relationship between ERα and ERβ [8], [9]. Considering that ERα has been identified an important role in malignancies by more and more studies, we thus proposed that upregulation of ERβ may inhibit the promotion of breast cancer. Here we focused on ERβ expression changes in MCF-7 cells after the treatment of calycosin, as well as the alterations in ERβ-mediated signaling pathway.

Insulin-like growth factor 1 receptor (IGF-1R) signaling participates in regulation of cell proliferation and apoptosis, and supports the development of both normal tissues and malignancy [10]–[12]. Recently, a number of studies have indicated that estrogen could interact with IGF-1R pathway via ERα, followed by increased proliferation, enhanced metastasis and reduced sensitivity to apoptosis [13], [14]. On the other hand, Tang et al. provide the first evidence for an interaction between ERβ and IGF-1R in lung cancer [15]. Remarkably, our previous findings showed that formononetin, another member of isoflavones family, successfully inactivated insulin-like growth factor 1 (IGF-1)/phosphatidylinositol 3-kinase (PI3K)/protein kinase B (Akt) pathway in MCF-7 cells, leading to inhibition of cancer cell proliferation [16]. Thereby the possibility has been raised that calycosin may work as inhibitors of IGF-1R signaling pathway through ERβ instead of ERα, followed by regulation of downstream targets.

In brief, together with the anti-proliferation effect of calycosin against breast cancer cells, we here explored the role of ERβ-mediated IGF-1R pathway in ER-positive cells, so as to better define the molecular mechanism of calycosin functions. The results showed that calycosin significantly caused decreased proliferation and apoptosis in ER-positive breast cancer cells but not in ER-negative cells. Moreover, this antitumor activity was correlated with upregulation of ERβ subtype, downregulation of IGF-1R, selective regulation of mitogen-activated protein kinase (MAPK) and PI3K/Akt pathways, and finally activation of Poly (ADP-ribose) polymerase 1 (PARP-1) cleavage.

## Materials and Methods

### Cell culture

Human breast cancer cell lines MCF-7, T-47D, MDA-231 and MDA-435 were all obtained from American Type Culture Collection (Manassas, VA, USA). The cells were maintained in RPMI 1640 medium with 10% fetal bovine serum (FBS) and incubated at 37°C with 5% CO_2_ in a humidified atmosphere.

### MTT assay

The anti-proliferation effect of calycosin against tumor cells was evaluated by MTT assay. Cells were plated into 96-well plates (5×10^3^ cells per well) for 12 h, next incubated with various concentrations of calycosin (0, 25, 50, 100 µM). Calycosin was purchased from Phytomarker Ltd (Tianjin, China). After 24, 48 and 72 h, cells were respectively analyzed for viability by MTT assay. The optical density (OD) was read at 490 nm using a microplate reader (Bio-Tek Instruments, Winooski, VT, USA).

### Flow cytometry assay

After treatment with calycosin (0, 25, 50 and 100 µM) for 48 h, cells were stained with Annexin V-FITC and propidium iodide (PI) for 30 min at room temperature. The percentages of early apoptotic cells were calculated by a FACS Aria flow cytometer (Becton Dickinson), and the early apoptotic cells is defined by Annexin V-FITC positive and PI negative.

### Western blot assay

Extracts of calycosin-treated cells were prepared in a lysis buffer. Then 40 µg protein samples were separately subjected to sodium dodecyl sulfate-polyacrylamide gel electrophoresis (SDS-PAGE), transferred onto polyvinylidene difluoride membrane (Millipore, Bedford, MA, USA), and blocked by Tris-buffered saline containing 5% non-fat milk. The proteins of interest were respectively labeled by incubation with the corresponding primary antibodies overnight (ERα 1∶500, ERβ 1∶500, IGF-1R 1∶500, ERK1/2 1∶1000, p-ERK1/2 1∶2000, JNK 1∶1000, p-JNK 1∶2000, p38 1∶500, p-p38 1∶1000, Akt 1∶1000, p-Akt 1∶2000, PARP-1 1∶1000, β-actin 1∶1000). Anti-ERα, anti-ERβ, anti-IGF-1R and anti-β-actin were purchased from Santa Cruz Biotechnology (Santa Cruz, CA, USA). All of the other antibodies used in this study were from Cell Signaling Technology (Danvers, MA, USA). Each band intensity was quantified using Image pro plus 5.02 software and normalized to the intensity of the loading controls.

### Statistical analysis

The data were shown as mean ± SD. Comparison of different treatments was performed with one-way ANOVA tests. And a p-value<0.05 was considered statistically significant.

## Results

### Calycosin inhibited proliferation of ER-positive human breast cancer cells

**Figure 1:**
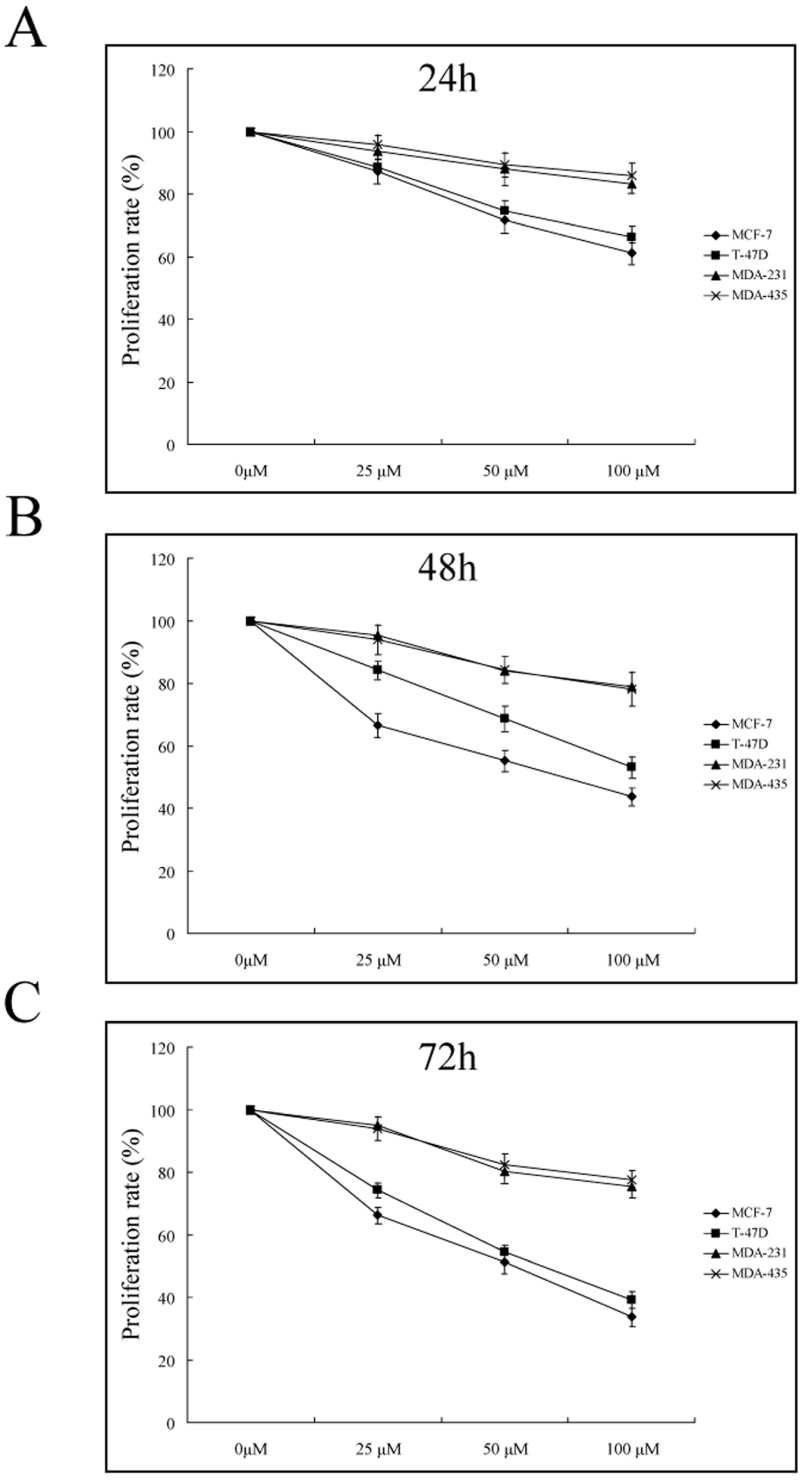
Inhibited proliferation of ER-positive breast cancer cells by calycosin. ER-positive cells (MCF-7, T-47D) and ER-negative cells (MDA-231, MDA-435) were separately incubated with varying concentrations of calycosin (0, 25, 50, 100 µM) for 24 (**A**), 48 (**B**) and 72 h (**C**). Then cell proliferation rate (relative to calycosin-untreated cells) was assessed by MTT assay. Results are representative of three independent experiments.

To reveal a potential difference of calycosin-induced anti-proliferation between ER-positive and ER-negative breast cancer cells, MCF-7, T-47D, MDA-231 and MDA-435 cells were separately incubated with calycosin (0, 25, 50, 100 µM) for 24, 48 and 72 h. Compared with ER-negative MDA-231 and MDA-435 cells, calycosin caused greater time- and dosage-dependent proliferation inhibition of ER-positive MCF-7 and T-47D cells, which suggested that calycosin mainly utilized ER-associated mechanisms to suppress tumor cell growth (Figure 1). Meanwhile, although calycosin induced similar growth inhibition in two ER-positive breast cancer cell lines, this effect was more obvious in MCF-7 than T-47D cells.

### Calycosin triggered death of ER-positive human breast cancer cells

Likewise, when breast cancer cells were treated with calycosin, no significant change in the percentage of early apoptotic cells was observed in MDA-231 or MDA-435 cells (Table 1). In contrast, calycosin elicited apoptosis in two ER-positive cells in a dosage-dependent manner, especially in MCF-7 cells that showed increased rate of apoptosis from 2.35±0.67% (0 µM) to 28.67±0.99% (100 µM). These observations, together with the results of MTT assay, indicated that activation of ER is involved in calycosin-induced growth regulation of breast cancer cells. Furthermore, to investigate whether IGF-1R and caspase-3 plays the critical role in this apoptosis, MCF-7 and T-47D cells were separately pre-treated with IGF-1R inhibitor (picropodophyllin, PPP) and caspase-3 inhibitor (Z-DEVD-FMK), followed by incubation with calycosin (100 µM) for 48 h. As shown in Table 1, the calycosin-induced apoptosis was prevented in part by inhibition of IGF-1R and caspase-3, suggesting that calycosin might be able to activate IGF-1R-mediated apoptotic pathway to induce cell death.

**Table 1:** Early apoptosis of human breast cancer cells quantified by flow cytometry (%).

Group	MCF-7	T-47D	MDA-231	MDA-435
0 µM	2.35±0.67	2.11±0.52	2.56±0.67	2.04±0.53
25 µM	5.34±1.23	4.68±0.78	3.42±0.45	3.21±1.23
50 µM	15.88±1.45**	11.92±1.34**	4.63±0.91	3.78±1.09
100 µM	28.67±0.99**	20.29±1.21**	4.59±1.01	4.11±0.89
100 µM+PPP	16.23±2.11**	12.11±1.54**		
100 µM+Z-DEVD-FMK	7.32±3.21**	6.77±2.34**		

** Compare with control group (0 µM) *p*<0.05.

### Upregulation of ERβ contributed to tumour-suppressor activities of calycosin

**Figure 2:**
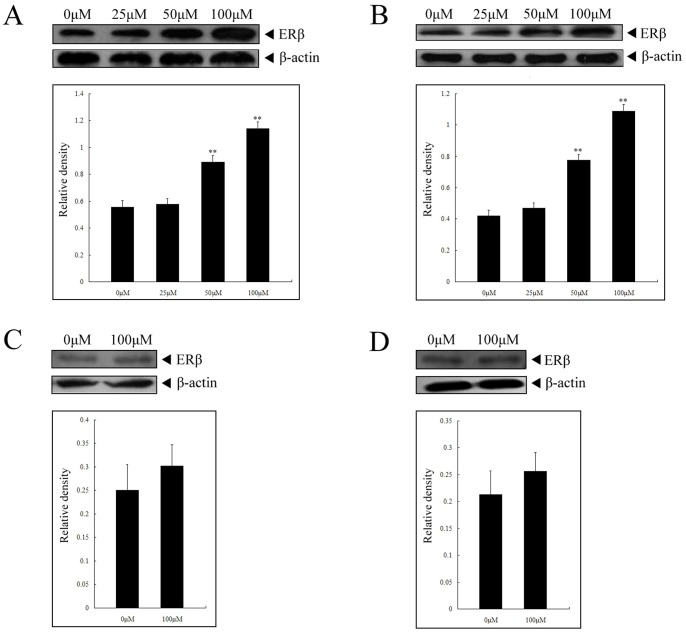
Upregulation of ERβ in ER-positive cells in response to calycosin treatment. MCF-7 (**A**), T-47D (**B**), MDA-231 (**C**) and MDA-435 cells (**D**) were separately treated with calycosin (concentration indicated) for 48 h. Cellular lysates were subjected to western blot assay using specific antibodies against ERβ. The same blots were probed with anti-β-actin antibody as a loading standard. Results are representative of three independent experiments. ** p<0.05 versus control group (0 µM)

Unlike ERα, the role of ERβ in human breast cancer is still uncertain, thus we focused our study on the effect of calycosin on ERβ. The results demonstrated that, in both MCF-7 and T-47D cells, the expression of ERβ was significantly upregulated with the increasing concentration of calycosin (*p*<0.05), as shown in Figure 2A and 2B. As for MDA-231 and MDA-435 cells, they are categorized as ER-, but we found they also expressed low levels of ERβ. However, calycosin did not alter the cellular levels of ERβ in MDA-231 and MDA-435 cells even when the concentration reached up to 100 µM (Figure 2C and 2D). Therefore, it could be deduced that the inhibitory action of calycosin on ER-positive breast cancer cells was achieved through increase in ERβ expression and subsequent regulation of ERβ signaling pathway.

### Calycosin inactivated IGF-1R signaling pathway in ER-positive human breast cancer cell

**Figure 3:**
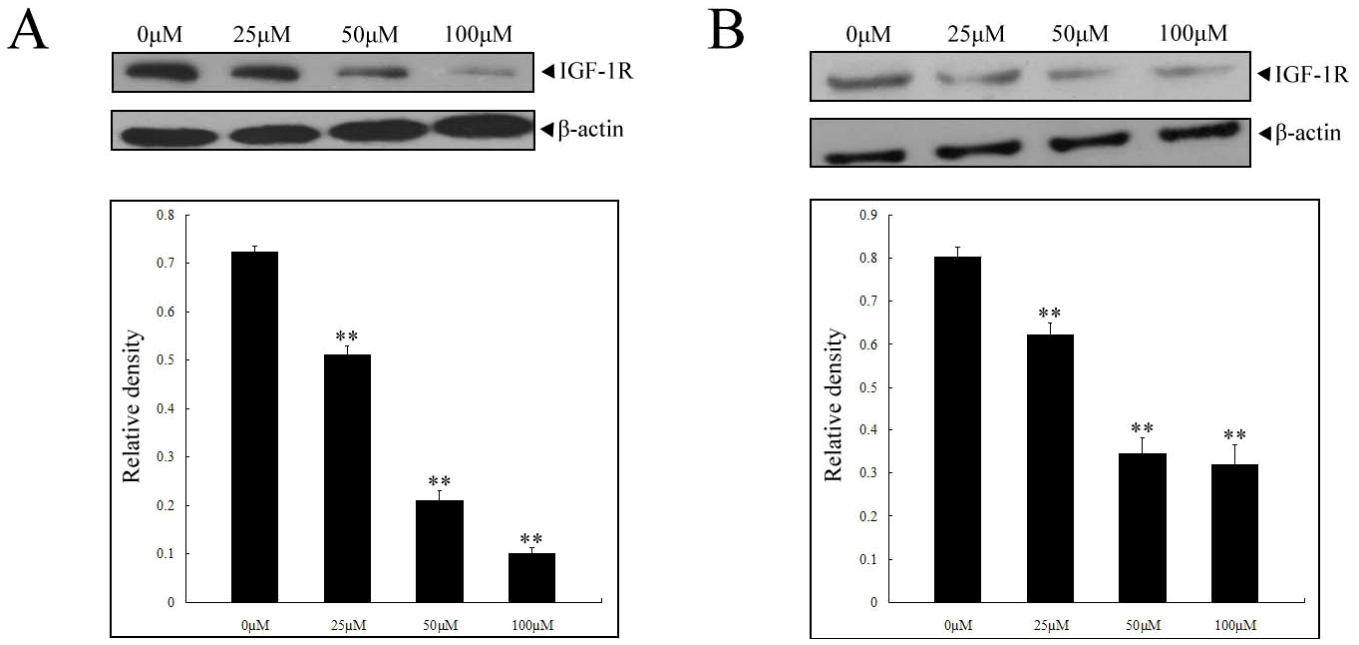
Downregulation of IGF-1R in ER-positive cells with treatment of calycosin. Western blot assay was performed in MCF-7 (**A**) and T-47D cells (**B**) (treated by calycosin as indicated for 48 h) to determine the expression of IGF-1R. Blots were probed for β-actin as loading controls. Results are representative of three independent experiments. ** p<0.05 versus control group (0 µM)

Epidemiological studies have associated breast cancer development with increased IGF-1 serum levels. Accordingly, inhibiting IGF-1R signaling may be a new strategy for the treatment of breast cancer. We next investigated whether increased ERβ would have a suppressive impact on IGF-1R signaling. MCF-7 and T-47D cells were respectively incubated with 0, 25, 50 and 100 µM calycosin for 48 h, and analyzed for expression of IGF-1R. As expected, IGF-1R was both highly expressed in ER-positive cells, whereas calycosin significantly reduced the protein levels of IGF-1R (*p*<0.05), especially at highest concentration (100 µM) in MCF-7 cells (Figure 3), which suggested that ERβ-directed downregulation of IGF-1R was involved in calycosin-mediated cell death.

**Figure 4:**
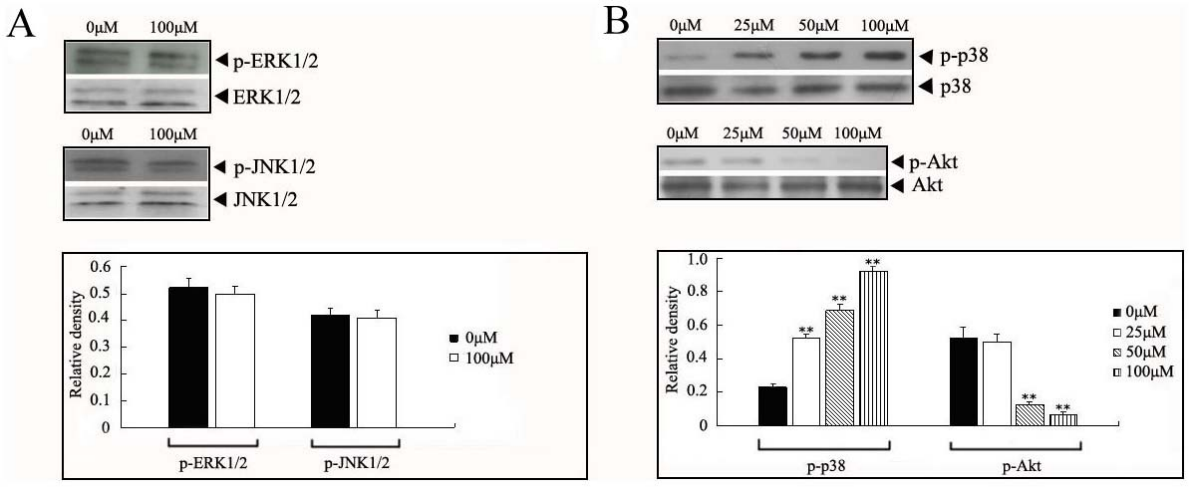
Selective regulation of MAPK and PI3K/Akt pathways by calycosin in MCF-7 cells. Cells were treated with calycosin (0, 25, 50, 100 µM) for 48 h. Then the activity of ERK1/2 and JNK (**A**) was determined using western blot assay, along with alteration of p38 and Akt activity (**B**). The antibodies to total ERK1/2, JNK, p38 and Akt served as loading controls. Results are representative of three independent experiments. ** p<0.05 versus control group (0 µM)

In view of the high sensitivity of MCF-7 cells to calycosin, it was then chosen to further identify the possible relationship between IGF-1R and downstream effectors. MAPK and PI3K/Akt is regarded as two main downstream pathways initiated by IGF-1/IGF-1R. Then, following IGF-1R detection, we respectively determined the activity of ERK1/2, JNK, p38 and Akt in calycosin-treated MCF-7 cells. For ERK1/2 and JNK, even high concentrations of calycosin (100 µM) showed negligible impact on their phosphorylation (Figure 4A). On the contrary, calycosin markedly enhanced p38 phosphorylation, but inhibited phosphorylation of Akt, both of which showed dose dependence (*p*<0.05), as shown in Figure 4B. Together, these findings imply that ERβ-IGF-1R signaling pathway may exert the opposite effect on activation of MAPK and PI3K/Akt in ER-positive cells.

### Calycosin induced cleavage of PARP-1 in ER-positive human breast cancer cell

**Figure 5:**
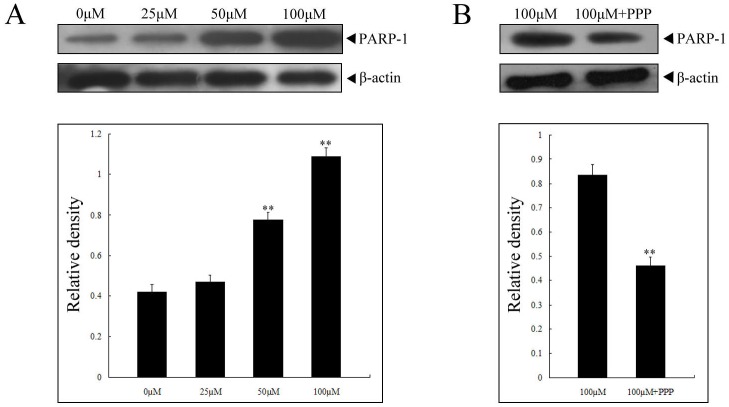
Increased cleavage of PARP-1 by calycosin in MCF-7 cells. After treatment with 0, 25, 50, 100 µM calycosin for 48 h (**A**), or IGF-1R inhibitor (PPP) prior to 100 µM calycosin (**B**), the cleavage of PARP-1 in MCF-7 cells was determined by western blot. The expression of β-actin served as loading controls. Results are representative of three independent experiments. ** p<0.05 versus control group (0 µM) or 100 µM group

Cell apoptosis is often correlated with cleavage of particular substrates such as the PARP-1 protein, the excessive activation of which could promote cell apoptosis. Here we treated MCF-7 cells with 0, 25, 50 and 100 µM calycosin for 48 h. The levels of cleaved PARP-1 were determined by a commercially available antibody specific for cleaved PARP-1. It was found that calycosin induced dosage-dependent cleavage of PARP-1 in MCF-7 cells (*p*<0.05), as shown in Figure 5A. Moreover, when MCF-7 cells were pre-treated with PPP, PARP-1 cleavage was significantly inhibited (Figure 5B), indicating that PARP-1 may be downstream target of IGF-1R, MAPK and PI3K/Akt pathways regulated by calycosin.

## Discussion

In the present study, we analyzed four available breast cancer cell lines MCF-7, T-47D, MDA-231 and MDA-435. The results showed that the treatment of calycosin failed to exert a potent inhibitory effect on proliferation in ER-negative MDA-231 and MDA-435 cells. Instead, calycosin led to time- and dosage-dependent induction of growth inhibition and apoptosis in ER-positive MCF-7 and T-47D cells. It is likely that whether or not calycosin participates in growth regulation of breast cancer cells would mainly depend on estrogen receptor. Here T-47D cells behaved similarly to MCF-7 cells, so both MCF-7 and T-47D cells were used to further study the correlation between calycosin and ER, with MDA-231 and MDA-435 cells as control.

Most previous studies have proved the interaction between estrogen/phytoestrogen and ERα in promoting cancer progression [17], [18]. By contrast, although increasing evidence from *in vitro* and *in vivo* studies supports that ERβ has tumour suppressive properties by inhibiting cell proliferation and triggering cell cycle arrest, the interaction between estrogen/phytoestrogen and ERβ is uncertain [19]–. Hence, we determined the expression levels of ERβ in breast cancer cells based on calycosin-induced antiproliferative activity. In the case of ER-positive cells (MCF-7 and T-47D), treatment of calycosin markedly elevated the levels of ERβ with drug concentrations. Nevertheless, even the highest of calycosin (100 µM) failed to significantly increase ERβ expression in ER-negative MDA-231 and MDA-435 cells. From these results, it can be concluded that calycosin induces the expression of ERβ to suppress cell proliferation, confirming the association between phytoestrogen and ERβ in breast cancer cells.

Following the demonstration of relationship between calycosin and estrogen receptor, a further step was to elucidate how activated ERβ signaling mediate these effects, especially the potential downstream effectors. IGF-1R, a heterotetrameric tyrosine kinase receptor, consist two α-chains and two β-chains [22]. After binding to IGF-1 and IGF-2, IGF-1R is phosphorylated and activates the downstream signaling pathway, culminating in proliferation and protection from apoptosis [23]. Increased expression of IGF-1R has been reported in many carcinomas including breast, prostate, pancreas and colon, and thus the blockade of IGF-1R activity can significantly induce growth inhibition and apoptosis in these cancer cells [24]–[26]. Besides, Kahlert et al reported that ERα could rapidly induce the phosphorylation of IGF-1R and the activation of downstream signaling cascades [27]. Therefore, given the antagonistic relationship between ERα and ERβ, we speculated that calycosin-mediated stimulation of ERβ might inhibit IGF-1R pathway, contributing to its antitumor effects. First, IGF-1R inhibitor PPP was applied to identify the involvement of IGF-1R pathway in calycosin-mediated apoptosis. In support of our hypothesis, when ER-positive breast cancer cells were pretreated with PPP, followed by calycosin incubation, the apoptosis was reduced in MCF-7 and T-47D cells with reduced apoptosis rates. Furthermore, as the expression of ERβ gradually increased with calycosin, the levels of IGF-1R in calycosin-treated MCF-7 and T-47D cells were observed to decrease in a dose-dependent manner, indicating the regulation of IGF-1R by calycosin through activation of ERβ.

The inactivated IGF-1R subsequently regulated kinase cascades, primarily MAPK and PI3K/Akt pathways. In mammalian cell, MAPK family consists of ERK1/2, JNK and p38 [28]. Generally, upregulation and activation of ERK1/2 and JNK tends to promote tumor development, whereas p38 acts as tumor suppressors. Acconcia et al. demonstrated that elevated expression of ERβ in HeLa cells resulted in a rapid activation of p38 and induction of cell apoptosis [29]. Similarly, we here found that more p38 was phosphorylated in response to calycosin-induced upregulation of ERβ in MCF-7 cells that was the most sensitive to calycosin treatment. However, the phosphorylation of ERK1/2 and JNK did not alter with calycosin over the time, meaning that there is no indication for the involvement of ERK1/2 and JNK in IGF-1R-mediated molecular mechanism of calycosin. On the other hand, it is reported that phosphorylation of IGF-1R could contribute to Akt recruitment and consequently its phosphorylation-dependent activation [30]. In present study, followed decreased expression of IGF-1R, the levels of phosphorylated Akt dropped with increasing concentrations of calycosin in MCF-7 cells. Overall, our findings indicate that the effect of calycosin on ER-positive breast cancer cells is achieved by ERβ-mediated selective regulation of IGF-1R/MAPK and IGF-1R/PI3K/Akt pathways.

We have known that activation of distinct caspase cascades plays a key part in apoptosis through cleaving some key proteins concerning cellular function and viability [31]. To confirm the activation of caspase-3 in apoptosis during calycosin treatment, Z-DEVD-FMK was used to block caspase-3 activity and caused reduced apoptosis in calycosin-treated ER-positive cells, suggesting that calycosin induced cell death via caspase-dependent pathway. One of caspase substrates is PARP-1 that has been implicated in a wide range of physiological functions, the most important of which is repair of damage DNA [32]. PARP-1 could be cleaved by caspase 3 into two specific fragments (89-kD and 24 kD, respectively). Importantly, 24-kD PARP-1 fragment is retained in the nucleus and attenuates DNA repair by blocking DNA repair enzymes, thus contributing to cell death [33]. The present study showed that calycosin finally promoted cleavage of PARP-1 in a dose-dependent manner in MCF-7 cells, which suggests that accumulation of cleaved PARP-1 finally results in calycosin-induced apoptosis. What's more, these changes in PARP-1 could be significantly attenuated by IGF-1R inhibitor PPP, further confirming that PARP-1 function as downstream targets of IGF-1R signaling pathway.

Together, our study provides further evidence for calycosin-mediated anti-tumor activity in ER-positive breast cancer cells, as well as the interaction of calycosin with ERβ. Meanwhile, its anti-proliferative effects are mainly mediated through ERβ-induced selective regulation of IGF-1R/MAPK and IGF-1R/PI3K/Akt pathways. These advances in understanding the pro-apoptotic mechanism may offer opportunities for clinical application of calycosin in treating breast carcinoma.
